# An Investigation of the Accuracy of EC5 and 5TE Capacitance Sensors for Soil Moisture Monitoring in Urban Soils-Laboratory and Field Calibration

**DOI:** 10.3390/s20226510

**Published:** 2020-11-14

**Authors:** Tala Kanso, Marie-Christine Gromaire, David Ramier, Philippe Dubois, Ghassan Chebbo

**Affiliations:** 1 Leesu, Ecole des Ponts, Universite Paris-Est Creteil UPEC, Champs-sur-Marne,77455 Marne-la-Vallee CEDEX 2, France; marie-christine.gromaire@enpc.fr (M.-C.G.); philippe.dubois@enpc.fr (P.D.); ghassan.chebbo@enpc.fr (G.C.); 2 Faculty of Engineering Third Branch, Lebanese University, Hadath Campus, 6573/14 Beirut, Lebanon; 3 Equipe projet TEAM, Cerema, 12 rue Teisserenc de Bort, 78190 Trappes, France; david.ramier@cerema.fr

**Keywords:** calibration methods, manufacturer calibration, soil storage, soil water content, sustainable drainage systems

## Abstract

Recently, emphasis has been placed on finding a reliable estimation of soil water content. In this study, two capacitance sensors EC5 and 5TE (METER Group) were utilized. These sensors provide many benefits relative to other sensors in that they are cost-effective and very economical regarding energy use, operate at a high measurement frequency of 70 MHz, and are dedicated to measuring at a small volume because of their small size. This makes them suitable for the context of use in this research, which consists of multiple sustainable drainage systems SuDS. Several studies have evaluated these two types of sensor but not for urban soils with specific characteristics. In addition, results from the literature are divergent and the published calibration data are limited. Therefore, an in-depth investigation of their accuracy is assessed in this paper. At first, the literature’s existing procedures and methods were examined. The sensor-to-sensor variability, as well as repeatability, were tested in soil and solutions. Additionally, a field calibration method was conducted to estimate the effects of soil texture on sensors readings. Two laboratory calibration methods having different principles were also applied, compared to each other and to the field calibration as well. Results revealed weak sensor-to-sensor variability (coefficient of variation up to 15% in soil) and also good repeatability (0.1%), for both sensors. A soil-specific calibration equation has improved the estimation of the volumetric water content. In case of soil having high field bulk density, the undisturbed soil calibration method described and proposed in this paper gives promising results. The latter method yields a volumetric water content (VWC) prediction accuracy of 0.025 m^3^∙m^−3^ on a sandy loam soil. This paper presents a large knowledge of capacitance sensors measurement technique as well as their calibration procedures and methods. Limitations of existing procedures have been identified and key elements for selecting the appropriate one are suggested. Derived calibration equations have also been provided for three urban soils with different particle size distribution, ranging from sandy loam to silt loam. Accurate monitoring of soil moisture content in urban soils is thus achievable.

## 1. Introduction

Precise measurements of soil moisture content in vadose zones remain a major challenge for estimating water balances, characterizing chemical and biological processes and also for developing hydrological models. A reliable and adequate measurement technique is therefore required to characterize the spatial and temporal variability of soil moisture. Existing techniques are classified into two different categories: the first requires contact with the soil while the second does not. Both of these have advantages but also limitations. For instance, remote sensing [1,2,3], a contact-free technique, enables large scale measurement and can be used in isolated location [4]. Yet it is highly affected by vegetation and surface roughness, and is limited to near-surface measures. To measure with high spatial and temporal resolutions at the point scale and in the soil profile, contact-based techniques are more appropriate.

Among contact-based techniques, time-domain reflectometry (TDR) [4,5], and capacitance sensors allow continuous soil water content monitoring [6,7,8,9]. TDR has been shown to be highly accurate since they operate at high frequency of 1 GHz, resulting in reduced salinity impact [10,11], yet their high price limits their application for large networks. Capacitance sensors operate at low frequencies from 10 to several hundred MHz [8], which makes them sensitive to soil environmental factors such as soil texture, bulk density, mineralogy, temperature, electrical conductivity, and supplied voltage [6,12,13,14]. Temperature effect on sensor readings is addressed by multiple studies [12,15,16,17]. The maximum variation in soil moisture reading estimated by a Hydra Probe sensor caused by a 40 °C change in temperature is equal to ± 0.028 m^3^∙m^−3^ [15]. On the other hand, the maximum error observed for the EC5 sensor at 40 °C and under saturated conditions is equal to +0.018 m^3^∙m^−3^ [12]. Bañón et al reported that the sensitivity of the EC5 and GS3 permittivity reading to temperature is highly dependent on soil salinity [16]. To illustrate, the effect of temperature on the soil moisture estimation by EC5 is negligible for a salinity below 10 dS∙m^−1^. In the present paper, temperature and salinity effects will not be investigated since the studied soil has lower salinity value (< 1dS∙m^−1^) and the temperature is within 20 °C.

Despite capacitance sensors sensitivity to many factors, they remain highly attractive and widely used in research applications, because of their low-cost allowing for a high number of measurement points, measurement ease, possibility of continuous data logging, and applicability to a wide range of soil [18,19]. 

Many of these sensors assess the relative dielectric permittivity of the bulk soil (see the review by [12]) which is then converted to volumetric water content through a calibration equation. Multiple studies have stressed the need of a site-specific calibration equation [19,20,21,22,23,24,25]. Manufacturers provide calibration equations. These equations has been occasionally recognized for its over-estimation [26], or under-estimation [20] of the actual water content, but also for having a lower precision than the specified ± 0.03 m^3^∙m^−3^ [19,27,28].

Sensors from the ECH_2_O series, EC-5 [29] and 5TE sensors [30] offer many improvements relative to other capacitance sensors, in that they are very economical regarding energy use, operate at a relatively high measurement frequency of 70 MHz [18], and are dedicated to measuring at a small volume because of their small size. This makes them suitable for the site studied in this research, which consists of multiple sustainable drainage systems SuDS, and for which a large sensor network with a maximum lifetime, a low sensitivity to the environmental factors, and a suitable sensor’s size that goes with the limited depth of medium is needed. SuDS are natural drainage management practices that aims to return the water cycle back to its natural regime by reducing runoff volumes and peak flows and improving water quality [31].

Accuracy of these sensors has been studied quite extensively, yet with conflicting results. The measurement accuracy of a reliable soil moisture sensor ideally suited for environmental research applications is ± 0.03 m^3^·m^−3^. At first, Kizito et al. has pointed out the accuracy of the EC5 and 5TE sensors, and determined a single calibration curve that can be applied on a range of mineral soils (sand, sandy loam, silt loam and clay), thus suggesting that there is no need for a soil specific calibration [18]. Still, other authors showed a significant sensor-to-sensor variability [24,32] and a soil characteristics dependency, by that affecting their accuracy. In order to guarantee the latter, evaluation and calibration of these sensors is thus needed. 

Two calibration procedures exist. The first one, called direct procedure, relates directly sensor output to the soil water content, while the second is a two-step procedure [33,34]. In numerous studies, the direct procedure is used to calibrate the EC5 [32] and 5TE sensors [21,28,35,36,37]. This procedure can be performed in the laboratory or in field. However, in field, it faces some challenges, such as covering a wide range of soil moisture contents.

In the two-step procedure, the first step, sensor output–dielectric permittivity relationship is determined by performing the electromagnetic (EM) sensor characterization method, using liquid standards [38]. In a second step, soil water content–permittivity relationship is established through an empirical model available for different soil types (e.g., [11]). There exist a number of studies that have performed this two-step procedure to calibrate EC5 [12,24] and 5TE [24] sensors, because of the ease of acquisition, the possibility to calibrate quickly a large number of sensors, and the avoidance of air gap and density variations during the first step. In practice and from a hydrological point of view, it is easier to interpret sensor output in terms of water content than in terms of permittivity. The accuracy of this interpretation is however dependent on the second step, which is the greatest source of in-situ calibration error [39]. The published equations for converting apparent dielectric permittivity to volumetric water content are derived in-situ on specific soils. Nevertheless, in the direct procedure, this accuracy is guaranteed, since specific in-situ soil characteristics are considered. 

Despite a large number of papers regarding capacitance sensors, little effort has been made to compare all contributed calibration equations and methods and limited published calibration data are available for EC5 and 5TE and for different ranges of mineral soils particularly in the case of soil in urban environment as those used for runoff water treatment. Urban soil constituting SuDS may have specific characteristics in terms of soil texture, type of minerals forming the mixture, mixing and compaction conditions, which can lead to high bulk densities, for example [40]. Continuous monitoring of soil moisture content in the SuDS is essential to assess its hydrological performance and understand water infiltration through the system. These data may be used to check the uniformly distributed inflow especially for linear SuDS or to detect the existence of preferential flow. Additionally, continuous monitoring of soil moisture content combined with a tensiometric sensor allows the determination of soil hydrodynamic characteristics such as the water retention curve. Soil moisture sensors combined with other water quantity measurement devices are used to calculate water balances as well. Moreover, very detailed monitoring data are required for the calibration and validation of hydrological models. 

Therefore, the objectives of the present paper are (i) to evaluate sensor-to-sensor variability and repeatability, (ii) to assess the accuracy of manufacturer calibration equation and the effect of soil texture on sensors output, for this purpose a field calibration was performed, (iii) to perform two different direct laboratory calibration methods and compare them with the field calibration, (iv) to compare manufacturer calibration with other direct/two-step calibration procedures reported in the literature, and finally, (v) to evaluate the uncertainty of prediction of soil moisture measurement. Soils used in this research are mineral urban soils with different textural classes (sandy loam, silt loam, and silt clay loam). 

## 2. Materials and Methods

### 2.1. Description of the Study Site and Instrumentation

**Figure 1:**
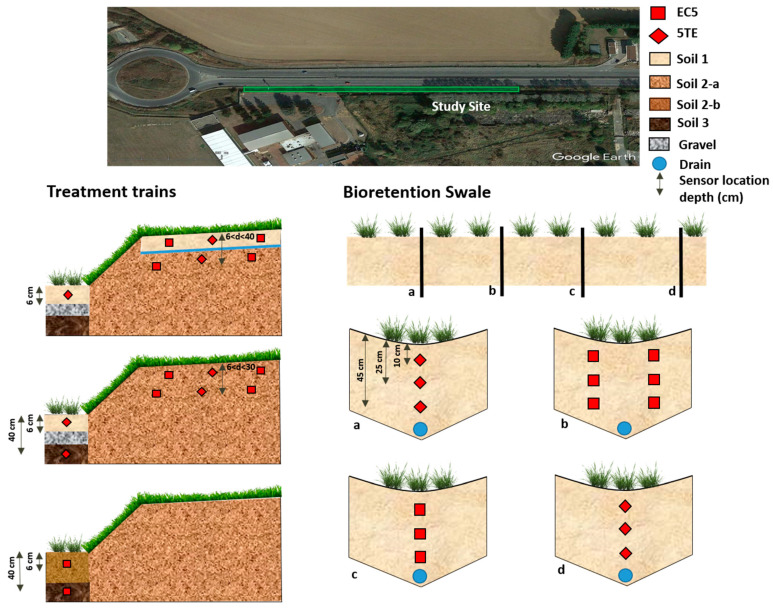
At the top a google earth photo of the studied site including the treatment trains and bioretention swale represented by a green rectangle. A schematic depiction (not to scale) of three treatment trains cross-sections is represented below the photo. A longitudinal section of the bioretention swale in addition to four cross-sections are also illustrated. The location and the installation depth of soil moisture sensors, the EC5 and 5TE, are indicated.

Sensors were tested on an experimental site, in Paris conurbation, where different sustainable drainage systems (SuDS) are implemented for road runoff management, including one bioretention swale and three treatment trains combining a vegetated filter strip with a bioretention facility. The number of sensors implemented on this site is of nineteen EC5 and thirteen 5TE, to continuously measure the water content in the soil. The bioretention facility has four equipped cross-sections, while each treatment train has one equipped section (Figure 1). 

Three types of soils constituting these SuDS have been studied. They present different textures ranging from sandy to clayey. For each soil, particle size distribution and bulk density were determined. Soil 1 is sandy loam [41], has 60.7% sand, 28.2% silt, and 11.1 % clay, and an in-situ bulk density of 1.6 g∙cm^−3^. The sand is basically calcareous sand. Soil 2 is silt loam, has 9.95% sand, 66.6% silt and 23.9% clay, and two different bulk densities 1.55 and 1.3 g∙cm^−3^, depending on soil location, they are referred in the following parts as soil 2a and 2b respectively. Soil 3, is a silty clayey loam, formed of 4.6% sand, 65.4% silt and 30% clay with a bulk density of 1.6 g∙cm^−3^. The number of EC5 sensors implemented in soil 1, 2a, 2b and 3 is 11, 6, 1 and 1, respectively. For 5TE this number is equal to 9, 3, 0 and 1, respectively. Near-surface sensors depth were chosen so as to have a minimum soil thickness to ensure the sensor’s measurement volume, thus the sensor is not exposed to surface conditions. The deeper sensors were installed to evaluate the vertical variability in SuDS. For instance, in the bioretention facility, three depths are chosen to estimate the soil moisture near-surface, in the middle and at the bottom of the system. The vertical spacing between the sensors ensures that there is no effect on each other. This difference in the number and location of sensors is related to pre-implementation strategy aiming to monitor the hydrological performance of these systems, compare various designs and finally validate hydrological models.

Information on the outputs and characteristics of the two sensors are listed in Table 1 including information from manufacturer manuals as well as the literature findings. The METER devices EC5 and 5TE capacitance-based sensors determine the apparent moist soil dielectric permittivity *ε*_a_, through measuring the charge time of a capacitor in the surrounding medium, at 70 MHz frequency [29,30]. They use an oscillator operating at a frequency of 70 MHz to measure dielectric permittivity. In addition to dielectric permittivity, the 5TE sensor having a three-prong design measures bulk electrical conductivity EC_b_ and temperature T. The volumetric water content (VWC) of the soil is determined through the dielectric permittivity *ε*_a_. For the EC5, a voltage is obtained at the output of the acquisition system, while for the 5TE the dielectric permittivity is directly acquired. The EC5 sensor is meant to measure volumetric water content from 0% to 100%, with an accuracy of 3%, at a temperature ranging from 0 to 50 °C. The 5TE sensor measures *ε*_a_, EC_b_, and T from 0 to 80%, 0 to 23 dS∙m^−1^ and −40 to 60 °C, respectively. The dielectric permittivity measurement accuracy in mineral soils of these sensors, as specified by the manufacturer, is ± 0.03 m^3^∙m^−3^. 

Power requirements are 2.5 to 3.6 V for the EC5 and 3.6 to 15 V for the 5TE sensor. The EC5 sensor has an analog output format that renders its output dependent on the supply voltage. However, the 5TE has a digital output format with no dependency on the supply voltage but rather on the communication mode between the sensor and the logger. Two communication ways are possible, the device driver interfaces (DDI) serial standard communication and the serial digital interface (SDI-12). At the studied site, EC5 and 5TE sensors were connected to non-METER data loggers, CR1000 and CR800 (Campbell Scientific, Logan, UT), at a supply voltage of 3.3 V, for data acquisition at a time step of 30 min continuously. Sensors were powered by a local battery and data was stored in data logger and then downloaded. The communication way is SDI-12 for the 5TE sensor.

**Figure 2:**
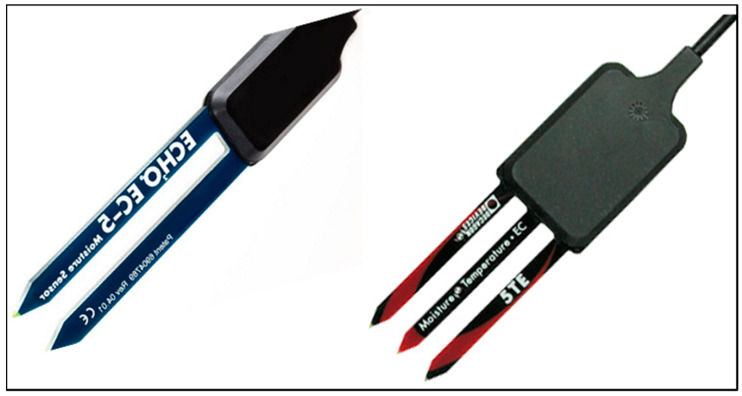
Prongs of the EC5 (**left**) and 5TE (**right**) sensors. The EC5 has two prongs while 5TE has three prongs. The length of these prongs is 5 cm.

The measurement volume, which is an ellipsoidal cylinder, is greater for the 5TE than for the EC5 (Table 1). This is due to the probe dimensions being 10 × 3.7 × 0.7 cm for the 5TE and 8.9 × 1.8 × 0.7 cm for the EC5 (Figure 2). A significant difference is found between the volume of influence provided by the manufacturer and that measured in other researches. For example, the sampling volume given by Sakaki et al. approximately corresponds to the volume of the sensor itself and is thirteen times smaller than the one provided by the manufacturer [32]. The latter has given a maximum possible volume of soil to avoid air voids on the sensor surface, thus ensuring an ideal measurement volume. Actually, the measurement volume depends on the nature of soil or material surrounding the sensor. In the manufacturer experiment the sensors were suspended in air where the electromagnetic waves are less attenuated than in soil. The measurement volume given by the manufacturer is therefore greater than the volume given by the other studies [36,42]. 

### 2.2. Sensor to Sensor Variability and Repeatability

At first, sensors were evaluated for their variability: (i) repeatability and (ii) sensor-to-sensor variability. The repeatability of sensor measurements was tested in-situ at a temperature of about 10 °C on a large number of sensors. For each sensor implemented in the field, 30 values were recorded, simultaneously for all sensors, over 30 min period with a 1 min time interval. The soil was rather under wet conditions. 

Sensor-to-sensor variability is linked to the production of these sensors, while repeatability is linked to their measurement precision. To evaluate sensor to sensor variability, nine EC5 and six 5TE sensors available in the laboratory at 20 °C were tested for 3 standards: air, water, and pure ethylene glycol, having a known dielectric permittivity of 1, 81.4 and 32.9 respectively. A single instantaneous measurement is recorded for each sensor. Additionally, the sensor to sensor variability was tested in soil during direct calibration procedure: for each volumetric water content, three EC5 and three 5TE were inserted in soils 1, 2a, 2b and 3. 

### 2.3. Sensors Calibration

#### 2.3.1. Overview of the Different Calibration Procedures and Methods

Different calibration procedures (direct/two-step) and methods (protocols) for the EC5 and 5TE are reported in the literature. The direct procedure directly relates the sensor output to the soil water content, while the second is a two-step procedure. In the first step, the relationship between the sensor output and dielectric permittivity is determined by applying the electromagnetic sensor (EM) characterization method using liquid standards [38]. In a second step, the relationship between soil moisture content and permittivity is established using an empirical model available for different soil types (Equation (1), [11]). Some of the reported calibration equations along with their accuracy in terms of volumetric water content were listed in Table 2. They are appropriate for the supply voltage and soil texture evaluated in this study.

(1) $$\theta \left(m^{3}\cdot m^{-3}\right)=-5.3\times 10^{-2}+\varepsilon_{a}\times 2.92\times 10^{-2}-\varepsilon_{a}{}^{2}\times 5.5\times 10^{-4}+\varepsilon_{a}{}^{3}\times 4.3\times 10^{-6}$$

As for the EC5 sensor, the manufacture direct calibration equation is given in Table 2. (Equation (2)). Additionally, Sakaki et al. two-point alpha mixing model (Equation (3)) is also provided [32]. This model proposed by Robinson et al. [43], needs only two sensor outputs corresponding to saturated and dry conditions to calibrate sensor for each soil. Thus, the time and effort needed for calibration are reduced. In this research, the two EC5 sensor outputs were measured in laboratory for each soil type. The calibration Equation (4) is derived from a two-step calibration procedure in [12]. In the first step, the EC5 sensor is calibrated through the standardized sensor calibration method proposed by [38] (using liquids with known dielectric permittivity). In the second step, usually, the empirical Equation (1) of [11] is applied to relate permittivity to soil water content, this equation is valid for a range of soil from sandy loam to heavy clay soils. In Bogena et al. study it has been found that the EC5 sensor output depends on the supply voltage, for this reason a permittivity–output voltage function (SRP model) was provided, for seven different voltages between 2 V and 5V [12]. Two sets of fitting parameters are presented, corresponding to 3 V and 3.5 V, since the supply voltage in this paper is 3.3 V.

Regarding the 5TE sensor, Equation (5) is the manufacturer’s equation obtained by a two-step procedure, during which the sensor is calibrated in air, glass beads saturated with ethylene glycol and pure ethylene glycol. In addition, Rosenbaum et al. Equation (6) [24], established with the same procedure as Bogena et al. [12] but for 5TE sensor is included. Lastly, a calibration Equation (7) resulting from a standard direct calibration method is also added [36].

#### 2.3.2. Calibration Methods Applied in this Study

##### The Standard Direct Calibration Method

The standard direct calibration method is a commonly used method proposed by Starr and Paltineanu [44] and recommended by the manufacturer in an application note by Cobose and Chambers [45]. This method consists of taking a disturbed sample in the field near the sensor location. Firstly, the sample is oven or air-dried in the laboratory, then incrementally wetted and sensor reading is recorded at each level of VWC. Other studies have calibrated the sensors following a similar direct calibration procedure on disturbed samples, however, the sample was incrementally dried rather than wetted [21,32,37]. In this study, EC5 and 5TE sensors were calibrated for soils 1, 2a, 2b and 3, using the above-mentioned manufacturer’s method. Calibration was carried out in the laboratory at a temperature near 20 °C. For each soil type, 4 L of soil were collected in the field near the area where the sensors measure. Soils were oven-dried at 105 °C for 24 h, then crushed to 2–5 mm, to allow for uniform packing layer by layer into a 5 L glass bulk soil container, with a bulk density approximately equal to the field bulk density. The volume of the container is chosen so that it is higher than the sensor measurement volume. Subsequently, sensors were inserted vertically into the soil, and reading was recorded using a Campbell Scientific data logger at a 3.3 V supply voltage. Three EC5 and three 5TE sensors were inserted one by one subsequently at each water content level. A known water volume was added to the soil to increase water content by 0.05 m^3^∙m^−3^. Then the soil was mixed homogeneously outside the container and repacked into it afterward. This process was repeated until the soil was completely saturated. At each water content level, a soil subsample was collected and volumetric water content was determined by gravimetric method [46].

##### Direct Laboratory Calibration on Undisturbed Sample

The direct calibration method may also be applied on an undisturbed soil core. Such a method was applied by Logsdon [47]: an undisturbed sample was taken from the site and then gradually wet to near saturation. It is however quite difficult to wet uniformly and progressively a soil core without disturbing it.

**Figure 3:**
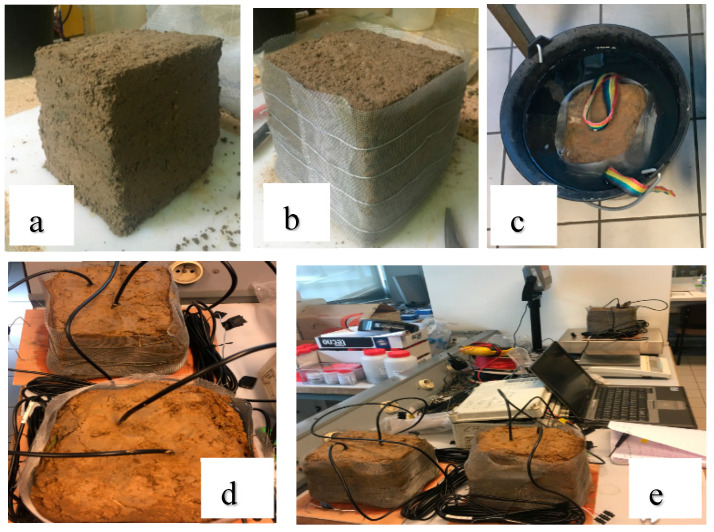
Undisturbed sample calibration method: (**a**) undisturbed block; (**b**) block packed with a metallic grid; (**c**) block put in water until saturation; (**d**) block allowed to dry in the open air; (**e**) taking measurements.

In our case, we choose to dry progressively an initially saturated undisturbed core of soil. Sensor readings are collected regularly during the drying process, and water content is evaluated in parallel by weighting the soil core. Soils for which EC5 and 5TE sensors were calibrated by this method, are soil 1, 2a, and 2b. Soil 3 was not studied here because of the difficulty of collecting an undisturbed sample of 4L at 0.5 m depth. For each soil type, an undisturbed block (Figure 3a) of approximately 4 L was collected in the field, packed with a metallic grid (Figure 3b) and then put in water for approximately 30 h until saturation (Figure 3c). EC5 and 5TE sensors were inserted vertically into the block, a first sensor reading was taken directly after saturation. The measurements were taken in the laboratory at a temperature of 22 °C. In order to decrease volumetric water content, the block was allowed to dry in the open air (Figure 3d–e), a reading was taken every two days, and the block was also weighted (mi). This process was repeated until the soil was completely dried (water content < 0.05 m^3^∙m^−3^). At the end of the experiment and after removing sensors, the block was oven-dried at 105 °C for 24 h, to determine the bulk density of the soil. At last the volumetric water content at each sensor reading was calculated. 

##### Field Calibration

Field calibration has been conducted by many researchers, wherein the volumetric water content of field soil is obtained through gravimetric method [46] and compared to the sensor output. It was found by [22], that the latter method yields to large error rates associated with collection of samples, voids, organic residues, and root densities, while [20,23,47,48] have recommended field calibration over laboratory and standard calibration provided by the manufacturer. Varble and Chávez [28] have also stated that a linear field-based calibration equation is satisfactory to achieve a high accuracy required in research applications.

In this study, field calibration was carried out to validate the standard and undisturbed direct calibration methods and to evaluate the effect of soil texture on sensor output. Field samples were taken close to the area where the sensors measure, at approximately the same depth, and at less than 30 cm from the sensor. This soil sampling was done during different periods, in order to cover a wide range of soil water contents. The in-situ temperature during the measurement period varies between 10 and 20 °C. Samples were collected using a sample auger (with an airtight soil-moisture cylinder). Like the undisturbed method, sensors in soils 1 and 2 were calibrated in field while soil 3 was excluded because of its location at a significant depth, which makes sample collection difficult. To determine the volumetric water content and field bulk density, soil samples were weighed, oven-dried at 105 °C for 24 h, and then reweighed. The calculated volumetric water content was then correlated with the sensor reading, for the half-hour during which the sample was taken (one measurement every 30 min). The resulting number of available field data, for each sensor type and soil type depends on the number and the location of sensors in-situ. The number of EC5 sensors implemented in soil 1 and 2 is 11 and 7, respectively. For 5TE this number is equal to 9 and 3, respectively.

### 2.4. Data Analysis 

Sensor-to-sensor variability and repeatability are evaluated through descriptive statistics, including the average value (Mean), and the dispersion described by the standard deviation (SD) and coefficient of variation (CV). 

Secondly, in order to verify whether a single calibration equation could be applied on all sensors of the same type, and for a specific soil, a statistical comparison test was conducted. Results obtained by the standard calibration method are used for this purpose. The three calibration data sets belonging to the same sensor type/soil type were compared within each other (two by two). This comparison test aims to determinate the existence or not of significant statistical differences between two data series, for which a linear regression has been fitted. At first, the residual variances $V_{1}$ and $V_{2}$ of the two series were compared by Fisher–Snedecor ($F_{test}$, Equation (8)). If the test reveals no significant differences between the two residual variances, then the slope a_1_ and a_2_ of the two series were compared using Student’s test ($t_{test}$, Equation (9)), with a risk α equal to 5%. This comparison continues in case of no significant differences between the two slopes, to test differences between the two intercepts b_1_ and b_2_ also using Student’s test (Equation (10)). Lastly, if tests concluded the equality of the slopes and intercepts, it is then possible to perform a common regression with all the values.

(8) $$F_{test}=\frac{V_{1}}{V_{2}}\;\mathrm{If}\;V_{1}\geq V_{2}\;;\;F_{test}=\frac{V_{2}}{V_{1}}\;\mathrm{if}\;V_{2}>V_{1}$$

(9) $$t_{test}\left(\mathrm{slope}\right)=\frac{a_{1}-a_{2}}{\sqrt{V_{c}{}^{2}\left[\frac{1}{\sum {\left(x_{1i}-\bar{x_{1}}\right)}^{2}}+\frac{1}{\sum {\left(x_{2i}-\bar{x_{2}}\right)}^{2}}\right]}}$$

(10) $$t_{test}\left(\mathrm{intercept}\right)=\frac{b_{1}-b_{2}}{\sqrt{V_{c}{}^{2}\left[\frac{1}{n_{1}}+\frac{{\bar{x_{1}}}^{2}}{\sum {\left(x_{1i}-\bar{x_{1}}\right)}^{2}}+\frac{1}{n_{2}}+\frac{{\bar{x_{2}}}^{2}}{\sum {\left(x_{2i}-\bar{x_{2}}\right)}^{2}}\right]}}$$

(11) $$V_{c}{}^{2}=\frac{V_{1}+{\;V}_{2}}{n_{1}+n_{2}-4}$$

With $V_{c}$ (Equation (11)) the common residual variance; $n_{1}$ and $n_{2}$ the sample size of the two series; $\bar{x_{1}}$ and $\bar{x_{2}}$ the means of the two series.

To test the effect of soil texture on the sensor output, field calibration points are compared to the manufacturer equation along with its ± 0.03 m^3^∙m^−3^ accuracy. 

Regarding the performed direct calibration methods, they were compared to each other. Linear and third-order polynomial adjustment are applied to each data set using the least square approach. To evaluate the goodness of the fit, the coefficient of determination (R^2^), and the root mean square error (RMSE) were calculated. 

The effect of calibration procedure is assessed in the following section by comparing the literature driven equations (Section 2.3.1) with each other and with those obtained in this study. Finally, 90% prediction intervals for the selected calibration models were provided for both sensors.

## 3. Results and Discussion

### 3.1. Repeatability and Sensor to Sensor Variability

In-situ replicate measurements for both sensors (Table 3) indicate good repeatability, since the maximum noticed coefficient of variation was 0.12 % of the output value, for 30 successive readings. Thus, this negligible repeatability is not a main source of variation for this type of sensors, as already shown by [24,32]. 

Results of the sensor to sensor variability in air, water and solution are presented in Table 4. For the EC5 and 5TE sensors, measurements indicate a larger sensor-to-sensor variability in air than in water and pure Ethylene Glycol. This is also the findings of [42] based on 10 EC5 sensor readings. Generally, in the three standards, this variability is smaller for the EC5 (CV ~ 1%) than for the 5TE sensor (CV > 7%). The 5TE sensor, who has as output the apparent dielectric permittivity, shows an approximately accurate measuring in air and pure ethylene glycol, whereas it underestimates this measure in water. To illustrate, $\varepsilon_{a}$ of water is theoretically equal to 81.4, while the average 5TE output for 6 sensors was equal to 70. 

**Figure 4:**
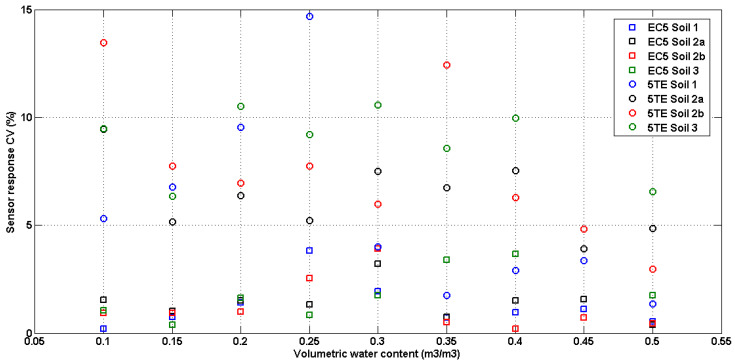
Scatter plot of the coefficient of variation (n = 3) of the volumetric water content measurement for two sensors, at different volumetric water content and in different soil texture. Results presented were obtained during the standard calibration method.

Sensor variability in the soil is represented in Figure 4. The coefficient of variation (CV) of readings from three different sensors in each soil type was scattered as a function of volumetric water content. For the EC5 the highest coefficient of variation is 4%, while for the 5TE sensors this coefficient is between 2 and 15%. Thus, the 5TE sensor-to-sensor variability in the soil is greater than the EC5 variability which was also demonstrated in air, water, and solution. Other studies have examined the variability of these sensors and found that sensor-to-sensor variability for 5TE is higher than EC5, for all VWC ranges and soil textures [24,49]. EC5 variability shows a tendency as a function of soil water content. For instance, the maximum CV for EC5 sensors corresponds to a volumetric water content of 0.25, 0.3 and 0.4 m^3^∙m^−3^, for soil 1, 2 and 3, respectively. It should be noted that soil 1, 2 and 3 are saturated approximately at 0.35, 0.45 and 0.5 m^3^∙m^−3^, respectively, indicating that the maximum variability falls in the near saturation range of VWC (~70% saturation). Kizito et al [18] and Rosenbaum et al. [24] have reported high variabilities in EC5 readings when the VWC is > 0.30 m^3^∙m^−3^, and an increase in sensor response scatter with increasing VWC which is not the case here. For 5TE sensors results show no significant difference in variability as a function of volumetric water content, since approximately for all VWC values this variation is > 5 %, and ranges maximums of 10% to 15% at different VWC depending on the soil type. In previous studies such as [24], this variability was tested only in solution because of the ease of acquisition, the avoidance of air gaps and density variations that may occur in soil and the possibility to separate the soil specific effect. On the other hand, it is important to examine the dependency of this variability on soil texture. Results in this study have revealed no significant effect of soil texture on sensor-to-sensor variability, for example the highest CV for the 5TE sensor at 0.1 and 0.25 VWC belongs to soil 2b and soil 1, respectively. Nevertheless, both sensors are more variable in silty clayey soil (soil 3) than in sandy loamy soil (soil 1) for all VWCs except 0.15 and 0.25. The effect of soil texture separately will be discussed in the following sections.

**Figure 5:**
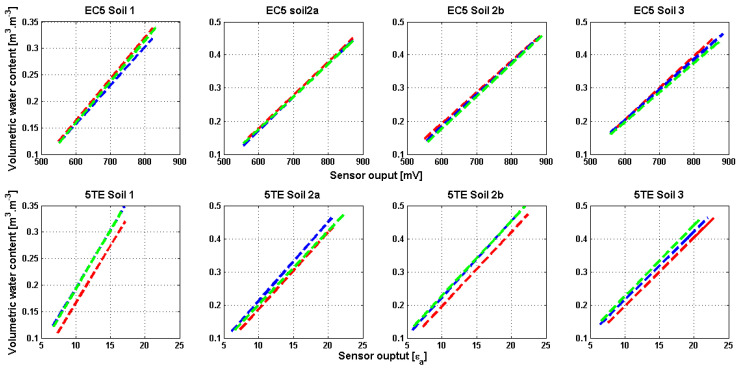
Linear regression equations obtained of the standard calibration method results, for the EC5 and 5TE sensor and for soils 1,2a, 2b and 3.

Sensor-to-sensor variability is therefore found to be dominant compared to the repeatability of the measurement. This brings into question, whether a single calibration equation could be applicable to other sensors of the same type. Accordingly, linear regressions for each sensor and each soil type were plotted in Figure 5 and statistically compared. Results confirm, as previously stated, larger differences in 5TE linear equations than in EC5. Nevertheless, the test concluded in all cases to the statistical equality (with a risk of 5%) of the slopes and intercepts of the three linear regressions. Rosenbaum et al. [24] in his study has revealed an increase in measurement accuracy of approximately 0.01 cm^3^∙cm^−3^ when a sensor-specific calibration equation is applied. The manufacturer findings confirmed an excellent sensor-to-sensor agreement, thus a single calibration equation may be applied to all sensors from one type and in particular soil. In general, and according to the results obtained in this study, the variability from one sensor to another is statistically acceptable. To decide whether a single equation is sufficient or not, several factors must be taken into consideration. The first factor is the accuracy required by the user which is related to the type of application. In the research context, for example the validation of physically-based hydrological model, good accuracy is crucial. Secondly, the number of sensors to be implemented also matters. If this number is large, calibrating each one is difficult and time-consuming. Finally, the choice of the sensor type is another factor since the EC5 sensor has better agreement in air, water, solution and soil than the 5TE sensor. This choice depends on the user needs for the additional parameters measured by the 5TE sensor.

### 3.2. Accuracy of Manufacturer Calibration

**Figure 6:**
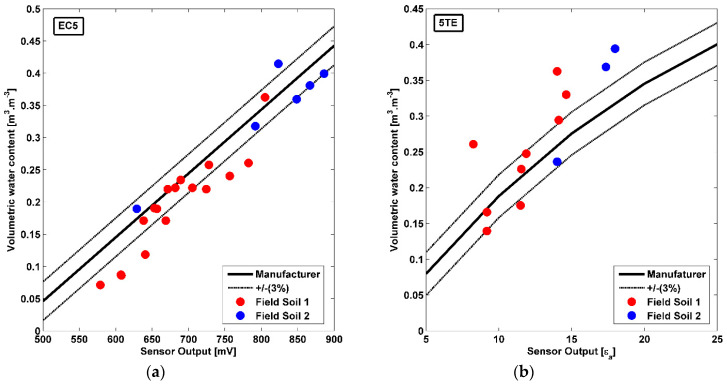
Field calibration results for the (**a**) EC5 and (**b**) 5TE sensors and for Soil 1 and 2 Comparison with the manufacturer equation (± 0.03 m^3^∙m^−3^ accuracy).

The results of field calibration for the two soil textural classes, sandy loam (soil 1) and silt loam (soil 2a and 2b) were plotted in Figure 6. The manufacturer calibration equations for EC5 and 5TE sensors (Table 2, Equations (1) and (2)), along with their ± 0.03 m^3^∙m^−3^ accuracy interval were also added. For the EC5 sensor, silt loam results are situated approximately within the manufacturer accuracy interval. However, numerous points of the soil 1 are situated outside this interval, particularly for VWC< 0.15 m^3^∙m^−3^ and around 0.25 m^3^∙m^−3^. Regarding the 5TE sensor, despite the small number of measurements (related to the number and location of sensors in-situ) for soil 2, results revealed good agreement with the manufacturer equation for VWC < 25 m^3^∙m^−3^. Beyond this VWC value, results are situated outside the interval for both textures. Generally, the manufacturer equation has been recognized for its over-estimation of VWC values [26], or underestimation [20]. Thus, a soil-specific calibration could improve sensors outputs and especially for 5TE sensor. Besides, the manufacturer recommends a soil-specific calibration equation for this type of sensors to increase their accuracy.

### 3.3. Comparison of Results from Different Direct Calibration Methods

**Figure 7:**
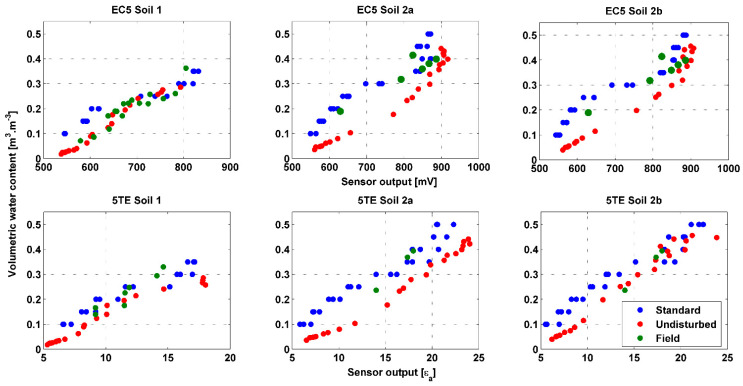
Sensors outputs plotted against the volumetric water content, obtained on the basis of direct calibration methods.

The three direct methods: standard, undisturbed and field are presented in Figure 7, performed on EC5 and 5TE sensors, for soil 1, 2a and 2b. Results show differences between the three methods. In the following text, a comparison between the standard and undisturbed method as well as an assessment of the goodness of each method will be discussed. The quality of the calibration method is deduced from its visual correspondence with the field-points. 

At first, the discrepancy between the methods is more noticeable for soils 2 than for soil 1, for both sensors. Especially, for the EC5 sensor in soil 2a, the discrepancy in VWC is up to 0.15 m^3^∙m^−3^. This may be related to the difference in procedure between the “undisturbed” and “field or standard” calibration methods. The soil moisture of the block in the undistributed method is probably not homogeneous, especially for soil 2 where the moisture gradient inside the block is likely to be more significant than for soil 1. Moreover, this is more likely when the soil dries out and the VWC < 0.3 m^3^∙m^−3^. Thus, inside the block the soil is more humid than the average water content of the block obtained by weighing. A better agreement is noticed between undisturbed method derived points and field points for soil 1. Figure 7 shows some field-measured calibration points (green dots) which are quite close to the points obtained in the laboratory with the undisturbed method (red dots). This is particularly remarkable for the EC5 sensor at VWC < 0.2 m^3^∙m^−3^. Regarding soil 2a and 2b the field points are closer to the standard calibration points, for both sensors. The undisturbed method was able to reproduce better the high field bulk density of soil 1 equal to 1.6 g∙cm^−3^. The effect of field bulk density is also noticed for soil 2 where soil 2a has a bulk density greater than 2b, respectively 1.55 and 1.3 g∙cm^−3^. To illustrate, the difference between standard and undisturbed methods for the same textural class is more likely to be significant for higher bulk density. According to Parsons and Bandaranayake, (2009), the EC5 sensor is very sensitive to changes in the bulk density of soil resulting from the different packings in the standard calibration procedure. Hence when the soil has high field bulk density, the undisturbed calibration method could be more adequate and yield to better results.

**Figure A1:**
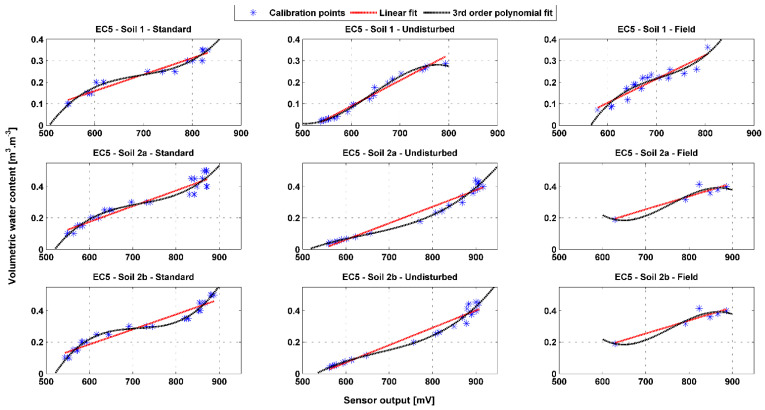
Linear and third-order polynomial fitting curves to EC5 sensor calibration data obtained by means of standard, undisturbed and field methods on soil 1, 2a and 2b.

**Figure A2:**
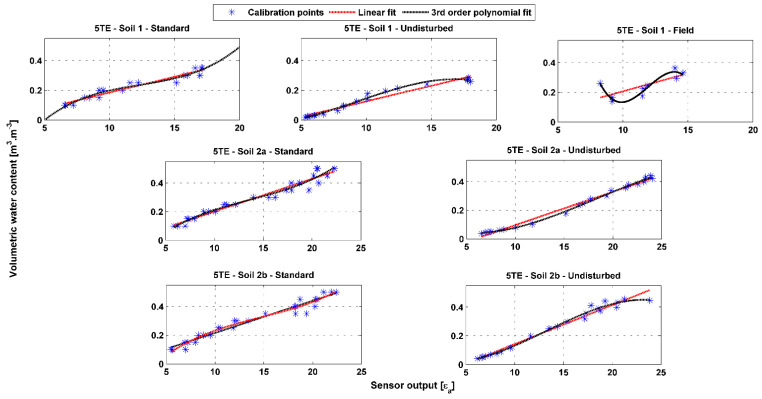
Linear and third-order polynomial fitting curves to 5TE sensor calibration data obtained by means of standard, undisturbed and field methods on soil 1, 2a and 2b.

Linear and polynomial third order regression parameters for the three direct calibration methods, are given in Table 5 for both sensor types, and for soil 1, 2a and 2b, along with their R^2^ and RMSE. Measurement points and fitting curves can be found for EC5 and 5TE in Appendix A, Figure A1 and Figure A2, respectively. Both fittings are significantly good, yielding to an R^2^ ~ 0.9 and RMSE < 0.03 m^3^∙m^−3^. The heterogeneity of field soil yields to more dispersed data, hence the lower R^2^ and RMSE values for field calibration.

The equations retained by this study are linear equations, since the transition to a 3rd degree polynomial model does not significantly affect the adjustment. Regarding the calibration method, field derived points were used to assess the quality of the other calibration equations. For soil 1, the undisturbed calibration method is retained, only for EC5 sensor. However, for the 5TE sensor, both standard and undisturbed equations were selected due to the difficulty of determining the one that best corresponds to the in situ points. Standard calibration equations were chosen for soils 2a and 2b.

### 3.4. Comparison of Direct and Indirect Calibration Procedures

Direct and two-step calibration procedures derived from the literature in addition to the calibration methods conducted in the present study are compared in this section for the EC5 and 5TE sensors. The calibration equations selected for soil 1 and soil 2 are those obtained by the linear undisturbed and linear standard direct methods, respectively. 

**Figure 8:**
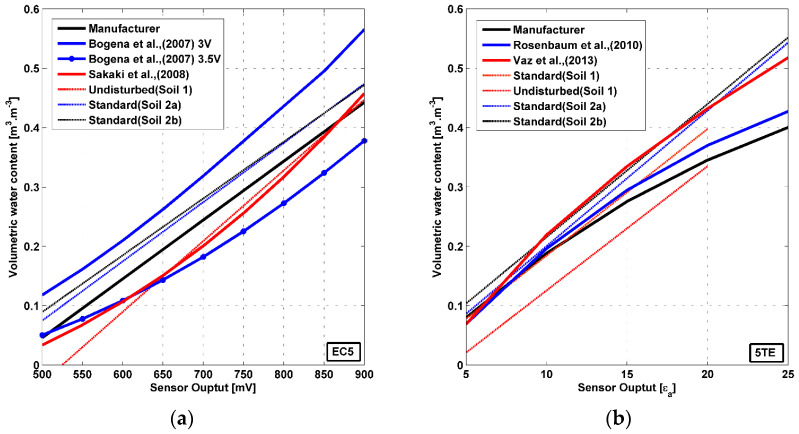
Comparison of different calibration procedures reported in the literature for the EC5 (**a**) as well as for the 5TE sensor (**b**).

For the EC5 sensor, the equations derived from two direct procedures, the manufacturer and [32] are plotted in Figure 8, in addition to the experimental soil specific calibration equations selected in Section 3.3. Two curves obtained by the two-step method conducted in [12] and corresponding to 3 and 3.5 supply voltage are plotted as well. These two latter equations show a large deviation one from the other and significant deviation from all the other curves. It should be noted that the supply voltage to which the other equations were conducted is equal to 3.3 V. Thus, the effect of supply voltage on the calibration equation is clearly identified here for the EC5 sensor. A good agreement is found between [32] model and the undisturbed equation for soil 1. However, the model by [32] underestimates the water content of the standard method for soils 2a and 2b by about 0.1 m^3^∙m^−3^. Soil 1 is a sandy loam soil (USDA) has 60.7 % of sand while soil 2 is a silt loam soil with 9.95% sand. Actually, the two-point alpha mixing model proposed by [43] and adopted by [32] was verified on industrial silica sands. Consequently, this model may provide reliable results but for soils with high sand content. The manufacturer equation systematically underestimation the VWC compared to the standard calibration values for soil 2, however the difference of about 0.03 m^3^∙m^−3^, remains consistent with the accuracy specified by the supplier. The accuracy is lower for sandy loam soils, according to Section 3.2 (Figure 6), to be around 0.06 m^3^∙m^−3^, especially at VWC < 0.25 m^3^∙m^−3^. 

Regarding the 5TE sensor, Figure 8 compares the experimental soil specific calibration equations selected in Section 3.3 to the curves derived from the manufacturer and [24] two steps procedures on the one hand, and the equation established by [36] with the standard direct procedure, on the other hand. The corrected apparent dielectric permittivity in [24] study does not show large improvement compared to the empirical equation of [11]. However the [36] direct calibration equation, corresponding to a limited range of mineral soils, shows discrepancies compared to the other two-step procedure equations. For instance, an apparent dielectric permittivity equal to 20 corresponds to 0.43, 0.37 and 0.35 m^3^∙m^−3^ with [36], [24] and the manufacturer equations respectively. Two-steps procedure equations also deviate from both our experimental soil specific standard and undisturbed methods equations. This raises questions about the reliability of the two-step procedure that applies an empirical equation and does not take into account soil texture. The experimental calibration curves obtained with the standard direct calibration for soil 2 a and 2b follow closely the direct [36] equation. For soil 1, the discrepancy between the latter equation and the standard calibration method equation is up to 0.03 m^3^∙m^−3^. This gap is approximately equal to the 0.025 m^3^∙m^−3^ precision given by [36] when applying the equation for mineral soils, with large textural variations (Sand, Sandy loam, Silt loam, and Loam). However, this is not the case for the undistributed calibration equation on soil 1 with a much higher discrepancy of 0.1 m^3^∙m^−3^. The manufacturer equation underestimates largely (> 0.05 m^3^∙m^−3^) the values obtained by the standard method for soil 2a and 2b for VWCs superior to 0.3 m^3^∙m^−3^. The direct (standard and [36]) and two-step procedures (manufacturer and [24]), with the exception of the undisturbed procedure, are relatively similar for the mid-range VWCs (0.2 < VWC < 0.3 m^3^∙m^−3^), but the difference increases near saturation. This emphasizes the effect of soil texture on calibration results, as well as the effect of soil bulk density. 

It should be noted that the calibration equations obtained for soils 2a and 2b are very similar to each other but are quite different from those of soil 1. This stresses the need for a specific calibration for each type of soil.

### 3.5. Evaluation of the Uncertainty of Prediction of Soil Moisture Measurement

**Figure 9:**
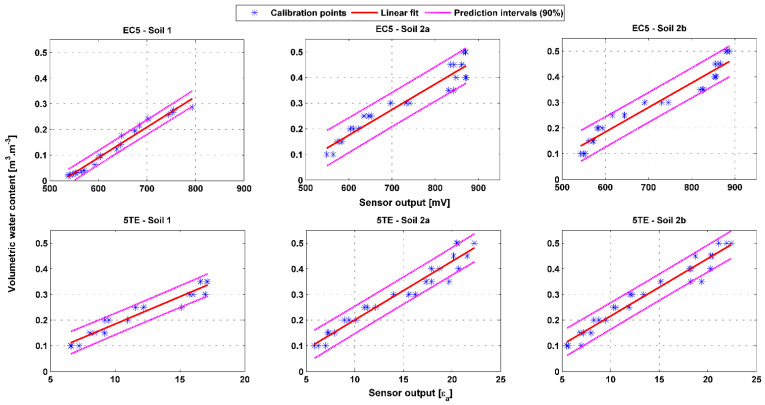
The 90 % predication intervals of the calibration models for sensors EC5 and 5TE in soil 1, 2a and 2b.

The uncertainty is essentially due to the uncertainty associated with the chosen calibration curve, as the measurement is well repeatable. Thus the 90% predictions intervals pertaining to the calibration models retained in this paper are presented in Figure 9. For both sensor types, the figures show greater uncertainty in the prediction of soil moisture value for soil 2a and 2b than for soil 1. This may be linked to the calibration equations provided in Section 3.3, Table 5. Notably, even with approximately equivalent R^2^, the calibration equation for soil 2 yields a greater RMSE than that of soil 1. For example, the calibration equation for the EC5 sensor, soil 1 and 2a, gives R^2^ values of 0.92 and 0.94, respectively. However, the corresponding RMSE is 0.022 m^3^∙m^−3^ for soil 1 which is less than 0.038 m^3^∙m^−3^ for soil 2. Comparison of the sensors with each other reveals that the prediction intervals are tighter for the EC5 than for the 5TE, particularly for soil 1. As for the latter, the predicted value falls, at a confidence level of 90%, within a range of ± 0.025 and ± 0.035 m^3^∙m^−3^, for sensors EC5 and 5TE (respectively). No significant difference from one type of sensor to another is noticed for soils 2a and 2b. The uncertainty of the prediction for this soil is approximately ± 0.045 m^3^∙m^−3^, for both sensors. It should be emphasized that even with a soil-specific calibration, the uncertainty on water content measurement with this type of sensor remains significant, and often greater than that given by the manufacturer. Nevertheless, the undisturbed soil method applied to sandy loam for which the moisture gradient of the soil block is relatively limited and for an EC5 with better sensor–sensor variability than the 5TE contributes to a promising accuracy of 0.025 m^3^∙m^−3^. 

## 4. Conclusions

Continuous monitoring of soil moisture in SuDS is often necessary for hydrological performance evaluation, water balance estimation, soil hydrodynamic characteristics determination and finally calibration and validation of hydrological models. Low-cost capacitance sensors are suitable for such type of use. Despite a large amount of research regarding capacitance sensors, little have compared all methods and equations and provided calibration data for the soils used in urban context. Urban soils may present specific characteristics such as high bulk densities.

Two low-cost capacitance sensors were deeply investigated in this study. The EC5 and 5TE sensors were implemented in a study site, which consists of multiple sustainable urban drainage systems (SuDS), including two treatment trains and one bioretention facility. They were tested for sensor-to-sensor variability and repeatability. In addition, the effect of soil texture on sensor outputs is evaluated through a field calibration method. Two laboratory calibration procedures were performed, compared to each other and to the field calibration as well. One of the methods proposed in this study, based on undisturbed soil cores, is appropriate for soils with high bulk densities that may be confronted in urban environment. Lastly, a comparison of different procedures and methods reported in the literature is assessed. 

The repeatability of both EC5 and TE sensor measurements is good, as the maximum noticed coefficient of variation was 0.1%. Generally, the 5TE sensor presents higher sensor to sensor variability than the EC5. This variability is greater in the air than in water, pure ethylene glycol, and soil. Yet, statistical analysis concluded that for a given soil, calibration curves are not significantly different from one sensor to the other, thus a single calibration equation is acceptable and can be applied for a given soil type and a given sensor type.

The manufacturer supplied calibration equation were developed for mineral soils with different textural classes. A comparison of this equation with field calibration points has been assessed. Results show that field points do not completely fit into the manufacturer calibration equation even if its ± 0.03 m^3^∙m^−3^ accuracy interval is taken into account. Thus, the application of a calibration equation to a wide range of soil texture class is not accurate, as demonstrated by the present study and other research. Therefore, a soil-specific calibration equation is crucial to obtain an accurate measurement of soil water content.

A direct calibration method relating directly the sensor output to the volumetric water content takes into account the effect of soil texture in the interpretation. Conversely, the two-step procedure, conducted in solution, may lead to significant errors by omitting the direct effect of soil texture. Manufacturer equation for the EC5 sensor seems more appropriate for silt loam soil than for sandy loam soil, as for the latter, the accuracy is > 0.06 m^3^∙m^−3^. The two-point alpha mixing model for EC5 proposed by [43] has led to promising results but for soils with high sand content. This model needs only two sensor outputs corresponding to saturated and dry conditions. Thus, the time and effort needed for calibration are reduced. For the 5TE sensor, the calibration equation provided by [36] suits better the soil with high silt and clay content. 

This study suggests the use of the direct procedure and a soil-specific calibration equation to obtain accurate measurements of water content. Among the direct procedures, the proposed the most reliable method is the field, although it is not always possible to collect samples in situ and to cover the complete range of VWCs. The standard method comes in the second place, except for very high density soils for which it is very difficult to reproduce the in-situ density in the lab on unstructured soil. The "undisturbed" method is a good alternative in the latter case, as long as the volume of the soil sample corresponds to the measurement volume of the sensor, so as not to have a bias linked to the gradient of the water content in the block of soil. The latter method applied on EC5 sensor yields a VWC prediction accuracy of 0.025 and 0.045 m^3^∙m^−3^ for sandy loam and silt loam soil, respectively. Further research is worth conducting to assess the effect of this uncertainty on the experimental evaluation of hydrological performance of SuDS as well as on the calibration and validation of hydrological models. 

## Figures and Tables

**Table 1:** Characteristics of the two sensors EC5 and 5TE.

	EC5	5TE
**Measurement range**	VWC (volumetric water content) (%): 0 to 100 T ( °C): 0 to 50	***ε*_a_**: 1 to 80 EC_b_ (dS∙m^−1^): 0 to 23 T ( °C): −40 to 60
**Number of prongs**	2	3
**Length, width, depth (cm)**	8.9,1.8,0.7	10,3.7,0.7
**Power supply**	2.5 to 3.6 V	3.6 to 15 V
**Output type**	**mV** (Non METER data logger) **RAW** = mV/0.732 (METER data logger)	***ε*_a_**, **EC_b_**, **T** (SDI-12 communication mode) ***ε*_RAW_, EC_b_, T_RAW_** (DDI serial) ***ε***_RAW_ = ***ε***_a_ × 50 T_RAW =_ 10 T + 400 (if T ≤ 50) ( °C) T_RAW =_ 2 T + 800 (if T > 50) ( °C)
**Output format**	Analog and dependent of the supply voltage	Digital and independent of the supply voltage
**Measurement volume (cm^3^)/diameter (cm)/** **Dimensions** **(axbxh cm)**	240 cm^3^/5.5 cm/ (5 × 5.5 × 9) (Manufacturer) 18 cm^3^ [32] 15 cm^3^ [42]	715 cm^3^/ 8.6 cm/(7 × 11 × 10) (Manufacturer) 79 cm^3^ [36]
**Accuracy VWC, EC_b_, T**	±3%	±3% ([11] equation), ±10%, ±1

**Table 2:** Calibration methods and procedures reported in the literature, with their equation and VWC accuracy. *θ* is the volumetric water content (m^3^∙m^−3^), x, x* and x¶ are the sensor output voltages (mV, V, and RAW = mV/0.732 respectively), $\varepsilon_{a}$ is the apparent dielectric permittivity.

Sensor	Equation No.	Source	Procedure	Method	Equation	Accuracy in VWC ( ± m^3^∙m^−3^) and R^2^
**EC5**	(2)	Manufacturer [29,30]	Direct	Standard calibration for mineral soils [44]	**2.5 V** supply voltage $\theta =11.9\times 10^{-4}\left(x\right)-0.401$ **3.3 V** supply voltage $\theta =9.92\times 10^{-4}\left(x\right)-0.45$	±0.03
	(3)	[32]	Direct	Long column drainage experiment using four industrial silica sands	$\theta =\frac{x{¶}^{\alpha }-x{¶}_{\mathrm{dry}}{}^{\alpha }}{x{¶}_{\mathrm{sat}}{}^{\alpha }-x{¶}_{\mathrm{dry}}{}^{\alpha }}\times \phi$ ϕ is the porosity of the soil α = 2.5 is the geometry factor	R^2^=0.973
	(4)	[12]	Two-step	First step: [38] method, liquids used are Dioxane (1,4-diethylene dioxide) and 2-isopropoxyethanol (i-C_3_E_1_) mixed with water. Second step: [11]	$\varepsilon_{a}\left(x^{*}\right)=\gamma +(1/\left[\alpha +(\beta /\left(x^{*}{}^{2}\right))]\right)$ Fitting parameters for a **3 V** supply voltage: α = −0.03128; β= 0.04148; γ(3 V) = −0.78628 Fitting parameters for a **3.5 V** supply voltage: α = −0.04173; β= 0.06855; γ(3.5 V) = −0.49469; $\theta =f\left(\varepsilon_{a}\right)$ [11]	±0.0178
**5TE**	(5)	Manufacturer [29,30]	Two-step	First step: Calibration in air, glass beads saturated with ethylene glycol and pure ethylene glycol Second step: [11]	***ε*_a_** = *ε*_RAW_/50 $\theta =f\left(\varepsilon_{a}\right)$ [11]	±0.03
	(6)	[24]	Two-step	Same procedure as [12]	$\varepsilon_{a}=0.0234\;\varepsilon_{RAW}-1.2917$	±0.010
	(7)	[36]	Direct	Standard calibration method for mineral soils bulk densities: 1–1.5 g∙cm^3^ Textural class ([41]): Sand/Sandy loam/silt loam/ loam	$\theta =-0.2945+0.1625\sqrt{\varepsilon_{a}}$	±0.026

**Table 3:** Statistics of the repeatability measurement of the two sensors in soil.

N = 30 Repeated Measurements for Each Sensor					
Sensor	Number of Sensors (N)	Soil	Mean per Sensor	SD per Sensor	CV (%)
			Min–Max (N Values)		
**EC5 (mV)**	11	Soil1	682–803	1.2 × 10^−13^–0.3	1.7 × 10^−14^–0.04
	6	Soil 2-a	716–868	0.06–0.37	0.01–0.04
	1	Soil 2-b	876	5.6 × 10^−13^	6.6 × 10^−14^
	1	Soil 3	875	0.07	0.01
**5TE (*ε*_a_)**	9	Soil1	12.5–16.8	1.1 × 10^−14^–0.016	6.4 × 10^−14^–0.12
	3	Soil 2-a	7.7–18.4	0–0.01	0–0.04
	1	Soil 3	18.8	0.01	0.04

**Table 4:** Statistics of sensors response in air, water, and pure ethylene glycol.

Sensor	Number of Sensors	Standards (Theoretical Value)	Mean Response (mV for EC5, *ε*_a_ for 5TE)	SD	CV (%)
EC5 (mV)	9 sensors	Air	344.2	3.5	1
		Water	1033.3	6.9	0.7
		Pure Ethylene Glycol	1001.5	5.8	0.6
5TE (*ε*_a_)	6 sensors	Air (1)	1.3	0.4	28.2
		Water (81.4)	70	5.7	8.2
		Pure Ethylene Glycol (32.9–37)	40.7	3.2	7.8

**Table 5:** Linear and third-order polynomial fitting parameters for the EC5 and 5TE sensors calibration data, obtained by the standard, undisturbed and field calibration methods (the parameters of the equations retained in the subsequent parts are highlighted in bold).

	Fitting Parameters θ=ax+b		R^2^	RMSE m^3^∙m^−3^	Fitting Parameters $\theta =ax^{3}+bx^{2}+cx+d$				R^2^	RMSE m^3^∙m^−3^
EC5 soil 1	a	b			a	b	c	d		
Standard	0.0007626	−0.2979	0.94	0.022	2.5 × 10^−8^	−5.2 × 10^−5^	0.036	−8.3	0.97	0.016
Undisturbed	**0.001192**	**−0.6255**	0.97	0.016	−2.8 × 10^−8^	5.3 × 10^−5^	−0.033	6.496	0.99	0.0097
Field	0.001104	−0.5576	0.86	0.029	4.2 × 10^−8^	−8.8 × 10^−5^	0.063	−14.95	0.89	0.027
**EC5 soil 2 a**										
Standard	**0.0009982**	**−0.4242**	0.92	0.038	2.4 × 10^−8^	−5.3 × 10^−5^	0.038	−9.096	0.94	0.035
Undisturbed	0.001051	−0.5691	0.95	0.034	8.7 × 10^−9^	−1.7 × 10^−5^	0.011	−2.537	0.99	0.019
Field	0.0008297	−0.3263	0.88	0.032	−3.9 × 10^−8^	9 × 10^−5^	−0.067	16.38	0.9	0.041
**EC5 soil 2b**										
Standard	**0.000957**	**−0.3892**	0.94	0.033	3.6 × 10^−8^	−7.8 × 10^−5^	0.056	−13	0.99	0.015
Undisturbed	0.001103	−0.591	0.96	0.034	1.3 × 10^−8^	−2.5 × 10^−5^	0.018	−4.183	0.98	0.024
Field	0.0008297	−0.3263	0.88	0.032	−3.9 × 10^−8^	9 × 10^−5^	−0.067	16.38	0.9	0.041
**5TE soil 1**										
Standard	**0.02131**	**−0.0284**	0.93	0.023	0.00033	−0.01221	0.165	−0.562	0.95	0.022
Undisturbed	**0.02095**	**−0.0838**	0.95	0.022	−0.00017	0.004784	−0.014	−0.019	0.99	0.011
Field	0.03134	−0.1415	0.9	0.024	−0.00057	0.02198	−0.248	1.01	0.91	0.029
**5TE soil 2a**										
Standard	**0.02285**	**−0.028**	0.95	0.030	0.000108	−0.00443	0.078	−0.238	0.96	0.03
Undisturbed	0.02327	−0.1361	0.99	0.018	−6.2∙10^−5^	0.003407	−0.033	0.134	0.99	0.01
Field	0.03962	−0.3184	1	0.0003	-	-	-	-	-	-
**5TE soil 2b**										
Standard	**0.02242**	**−0.0084**	0.95	0.03	0.000104	−0.004594	0.085	−0.263	0.96	0.03
Undisturbed	0.02728	−0.1309	0.97	0.026	−0.00011	0.004399	−0.025	0.054	0.99	0.019
Field	0.03962	−0.3184	1	0.0003	-	-	-	-	-	-
